# Diagnostic riddle– case report of ocular syphilis

**DOI:** 10.1186/s12348-025-00488-4

**Published:** 2025-03-26

**Authors:** Marta Bociąga-Kożuch, Aleksandra Raczyńska, Dorota Trela, Aleksander Garlicki, Tomasz Berus

**Affiliations:** 1 Ophthalmology Clinic, 5th Military Research Hospital and Polyclinic in Kraków, Kraków, Poland; 2 Department of Infectious Diseases, University Hospital, Krakow, 30-688 Poland; 3 Department of Infectious and Tropical Diseases, Jagiellonian University Medical College, Krakow, 30-688 Poland

**Keywords:** Syphilis, Ocular, Uveitis, Case report

## Abstract

Syphilis is one of sexually transmitted infections (STIs). The incidence of Treponema pallidum infection has increased in the last 20 years. This rise is also evident in ophthalmological practice, with cases of ocular syphilis becoming more frequent.

We present a case of a 29-year-old patient with blurred vision in his left eye. Patient showed no general symptoms, nor previous history of eye disorders. On ophthalmological examination, the best-corrected visual acuity (BCVA) was 20/20 in the right eye (OD) and 20/80 in the left eye (OS). The left eye presented high intraocular pressure (IOP) of 31 mmHg and symptoms of anterior uveitis with a linear branching corneal erosion. A B-scan ultrasound of the left eye revealed no vitritis. The preliminary diagnosis of herpetic infection was made, and antiviral therapy was introduced. Despite the initial improvement, symptoms of active anterior uveitis were found on follow-up visits. After approximately 4 weeks of ambulatory treatment, the patient was admitted to the hospital because of roseolae and lumps of the left iris, which appeared accompanied by a rash on patients’ lower limbs. Laboratory tests confirmed syphilis and human immunodeficiency virus (HIV) coinfection. During hospitalization intravenous treatment with penicillin and antiretroviral drugs was introduced. Therapy with penicillin was continued to 21 days with improvement in examination. On a follow-up visit after 6 months BCVA in both eyes was 20/20.

It is crucial to consider testing for STIs, especially Treponema pallidum infection, in the diagnostic process of patients with nontypical or nonresponsive to treatment ocular symptoms.

## Background

Syphilis is a bacterial infection caused by spirochaete *Treponema pallidum*. The spirochaete is transmitted mainly through sexual contact, but there are also rare cases of vertical transmission from mother to child during pregnancy. Transmission via blood products and organ donation is extremely rare due to donor screening tests for infectious diseases including syphilis.

The clinical manifestation depends upon the stage of disease, there is early (within the 12 months from infection) and late (1 year after infection) stage. The incidence of syphilis has increased in the last two decades. In the European Union rates of syphilis have shown an overall increase since 2000, which is caused mostly by an increase in Western and Central European countries and especially among men who have sex with men (MSM) [1].

Ocular syphilis is relatively rare, but overall increase of syphilis diagnosis indicates that the ocular syphilis can appear more frequently in everyday medical practice.

## Main text

### Case report

A 29-year-old male with no history of chronic diseases presented to the Emergency Department with blurred vision in his left eye for approximately 7 days without any systemic symptoms. He had no previous history of eye disorders nor injuries.

On ophthalmological examination, the BCVA OD was 20/20 and 20/80 OS. He had IOP of 18 mmHg OD and 31 mmHg OS, which after topical timolol, brimonidine and dorzolamide decreased to 22 mmHg. Examination of the left eye showed ciliary flush, corneal edema and keratic granulomatous precipitates, 2/3 + anterior chamber (AC) flare, iris rubeosis and irregular pupil with posterior synechiae. After staining with fluorescein, a linear branching erosion was found in the nasal-inferior quadrant of cornea OS. A similar, but smaller corneal erosion was found OD. The biomicroscopy OD was also notable for mild granulomatous precipitates in inferior part of cornea, but AC was clear.

Corneal edema and AC flare prevented a view of the left fundus and the B-scan ultrasound OS revealed no vitritis. Examination of the right fundus showed no abnormalities.

A subconjunctival injection with adrenaline was administered to the inferior fornix of the left eye with partial release of posterior synechiae.

Due to a suspected diagnosis of a herpetic infection of the eye, initial therapy with acyclovir orally and topical ganciclovir gel was introduced. In addition, mydriatics (tropicamide), steroid (dexamethasone) and hypotensive eye drops (timolol, brimonidine and dorzolamide), were prescribed.

After 10 days of treatment there was improvement in the patient’s vision: BCVA OS was 20/32.

Improvement was also apparent in the anterior segment: less staining with fluorescein, no linear branching erosion, 1 + AC flare. After another 10 days of topical treatment, there was further improvement in vision, BCVA OS reached 20/20, there was still mild active anterior uveitis, but also with obvious improvement.

On the follow-up visit after one week, although BCVA was still 20/20 in both eyes and IOP within normal range (15 mmHg OD and 20 mmHg OS), roseolae and lumps of left iris were found.

A few days previous to the appointment, the patient noticed a rash on his lower limbs, which rapidly worsened (Picture 1. Skin lesions on the patients’ thigh).

**Picture 1.**
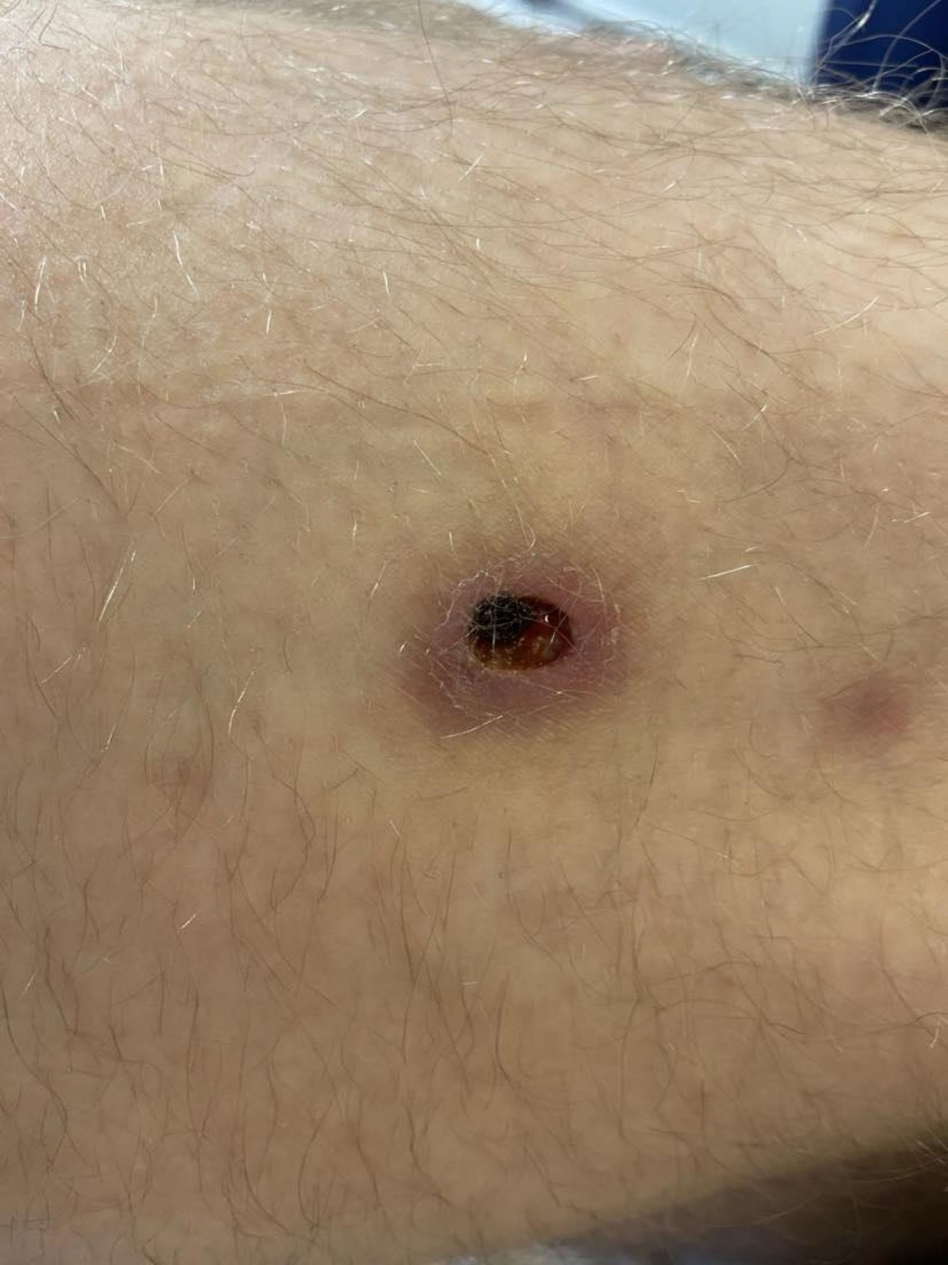
Skin lesions on the patients’ thigh

Because of skin changes and worsening of anterior uveitis patient was admitted to Ophthalmology Clinic and diagnostics for possible infectious cause were performed, including syphilis and Human Immunodeficiency Virus (HIV). We also checked for hepatitis B and C because of history of high risk sexual behaviors.

Also rheumatological diagnostics were considered and anti-nuclear antibodies (ANA), anti-neutrophil cytoplasmic antibodies (pANCA, cANCA) requested. Because of additional skin lesions associated systemic, rheumatological illness was considered such as vasculitis or inflammatory bowel disease.

Within 2 days AC OS findings got worse (Picture 2. Iris of the patients’ left eye with roseolae and lumps) and appeared also in the iris of the right eye.

**Picture 2.**
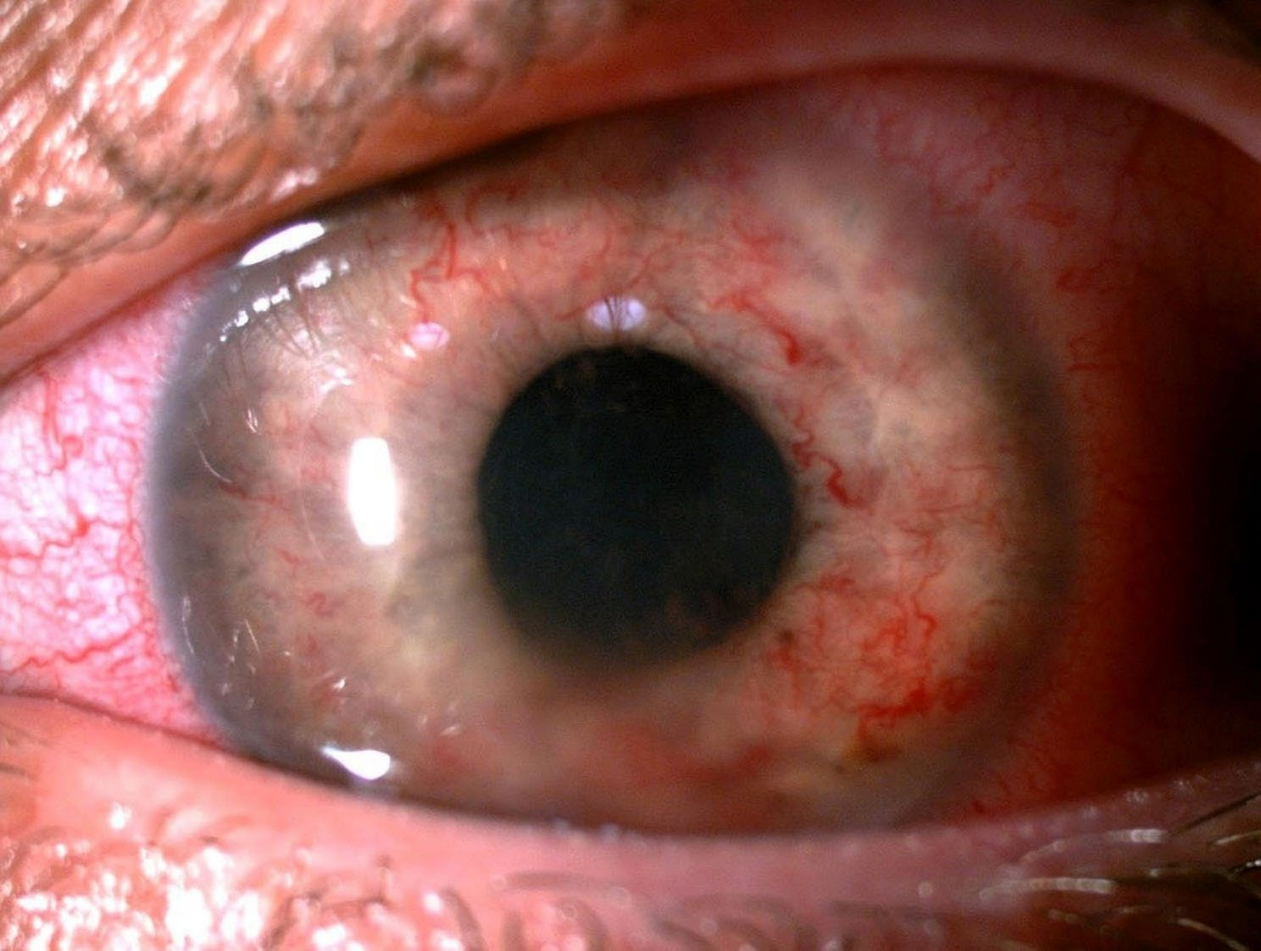
Iris of the patients’ left eye with roseolae and lumps

Ancillary testing revealed a positive fourth-generation HIV test (anti-HIV/p24) and positive serology for syphilis (rapid plasma reagin (RPR) = 1/128, treponema pallidum hemagglutination assay (TPHA) - positive). Following the positive screening test for HIV-1, a confirmation test was performed, detecting an HIV-1 RNA viral load of 20,500 copies/mL and a CD4 + count of 599 cells/µL at diagnosis. The patient was referred to the Infectious Disease Department to conduct further investigation and exclude central nervous system involvement. Intravenous treatment with penicillin and antiretroviral drugs (bictegravir + emtricitabine + tenofovir alafenamide) was introduced.

A lumbar puncture was performed without complication and cerebrospinal fluid (CSF) showed pleocytosis was 99 cells/L, CSF protein concentration was 1,28 g/L. The CSF-venereal disease research laboratory test (VDRL) was done: CSF-VDRL was nonreactive, CSF-TPHA was reactive. Biopsy of skin changes on the legs was performed, and histopathological examination revealed erythema induratum. Treatment with intravenous penicillin was continued to complete 21 days with progressive improvement on ophthalmological examination.

On a follow-up visit 1 week after discharge, BCVA was 20/20 and IOP was 8 mmHg, in both eyes.

On examination of the right eye singular, pigmented keratic precipitates in the inferior part of the cornea were visible; OS showed pigmented keratic precipitates in the paracentral and inferior cornea, minor iris rubeosis, irregular, wide pupil with posterior synechiae and pigment deposits on the lens. Local improvement and regression of skin changes of the legs were observed. After 6 months, BCVA in both eyes was 20/20, examination OS showed findings comparable to the previous examination.

On follow-up visits at the Outpatient HIV Clinic, laboratory tests revealed that the HIV treatment was effective, with an undetectable viral load after two months of therapy. Additionally, serological testing for syphilis (RPR) decreased to 1/32, demonstrating a positive response to penicillin therapy.

## Discussion

Rates of syphilis have increased significantly in the last several years in Europe. Between 2012 and 2021 they increased with a peak registered in 2019. After a decrease in 2020, the overall rate of syphilis cases in Europe increased again in 2021, reaching 7.0 cases of syphilis per 100 000 population [2]. 

In Poland, a similar trend in syphilis rates has been observed in recent years. Although during COVID-19 pandemic, in 2020, syphilis rates decreased, in 2021 they increased again. In 2020, there were 1,79 cases of syphilis per 100 000, in comparison to 2019 with 3,96 per 100 000 [3]. According to the European Centre for Disease Prevention and Control (ECDC), in 2021 the rate of syphilis in Poland was 3 cases per 100 000 [2, 3]. This decrease in 2020 can be caused by limited access to healthcare and change in habits during the COVID-19 pandemic [2–4]. Syphilis is often associated with other STIs. HIV co-infection is common, and it was found in 15% of reported cases [2].

The rise in the incidence of syphilis could be associated with rates of syphilis cases rising rapidly among MSM. The global syphilis prevalence among MSM was 7,5% over 20 years (between 2000 and 2020); prevalence estimates of syphilis among MSM, were higher in 2015–20 in Europe and Northern America in comparison to the period between 2010 and 14 [4].

Ocular syphilis can be present in all stages of the disease and syphilis can affect all parts of the eye. The most common diagnosis in ocular syphilis is panuveitis (41,3%) [5]. Other possible manifestations are anterior uveitis, intermediate uveitis, posterior uveitis with optic nerve involvement and/or retinitis/chorioretinitis [5, 6, 7]. Less common presentation form of ocular syphilis is isolated anterior uveitis (9,5%), but it is more likely to be found in HIV-positive patients [7]. 

In this case ocular syphilis presented initially as bilateral anterior uveitis branching treelike corneal erosions and high IOP in left eye. That led to the hypothesis of herpetic keratitis. Although unilateral presentation of herpetic uveitis is more common, bilateral involvement is mentioned in literature [8, 9], in 5% of patients [10]. In diagnostics of viral uveitis PCR-based analysis of aqueous humor sample should be considered, but a negative PCR result cannot exclude a herpes infection [9]. 

Bilateral anterior uveitis should be investigated to rule out infectious and non-infectious etiology, including syphilis. Serological tests are the method of choice for diagnosing Treponema pallidum infection. The non-treponemal tests (VDRL, RPR), which detect non-specific antibodies, and the treponemal tests (TPHA, treponema pallidum particle agglutination assay (TPPA), fluorescent treponemal antibody absorption (FTA-abs)). Non-treponemal tests can be used to monitor disease activity and treatment efficiency. Treponemal tests stay positive for life and are used as confirmation of the diagnosis, because they detect specific antibodies against Treponema pallidum bacteria [11]. 

The treatment for ocular syphilis is the same as for neurosyphilis. The first-line therapy is 18–24 million units daily of intravenous benzylpenicillin, as 3–4 million units every 4 h for 10–14 days. If benzylpenicillin therapy is not possible, there is an option of ceftriaxone or procaine penicillin and probenecid therapy [11, 12]. 

If there is anterior segment inflammation, topical steroids and mydriatics are indicated, in conjunction with penicillin treatment. Additionally oral steroids can be used, but their beneficial influence in therapy of ocular syphilis is not proven [12]. However, such treatment may be considered especially in cases of severe vitritis.

Treatment for HIV positive patients should be the same as for non-HIV-infected patients, although conducted with careful follow-up [11, 12]. The need for lumbar puncture in all cases of ocular syphilis is debatable. It is recommended by CDC to perform a lumbar puncture to examine for neurosyphilis only in individuals with ocular syphilis and other neurologic abnormalities, CSF analysis is not necessary in patients with isolated ocular symptoms. Patients with HIV infection have higher risk of CFS abnormalities, which are not necessary caused by neurosyphilis [12]. 

## Conclusions

The increased incidence of ocular syphilis could be an implication of the increasing rates of syphilis in general and growing knowledge among physicians to consider syphilis as a differential diagnosis in uveitis. It is crucial to take under consideration Treponema pallidum infection in diagnostics of patients with nontypical or nonresponsive to treatment eye symptoms. We presented, with photographic documentation, the case of iris roseola, that may be encountered in syphilitic uveitis. Recognizing these lesions may be helpful in diagnosing and treating ocular syphilis.

## Data Availability

No datasets were generated or analysed during the current study.

## Abbreviations

STI: sexually transmitted infection
BCVA: best-corrected visual acuity
OD: right eye
OS: left eye
MSM: men who have sex with men
IOP: intraocular pressures
CMV: cytomegalovirus
HIV: human immunodeficiency virus
AC: anterior chamber
ANA: anti-nuclear antibodies (ANA)
pANCA, cANCA: anti-neutrophil cytoplasmic antibodies
RPR: rapid plasma reagin
TPHA: Treponema pallidum hemagglutination assay
CSF: cerebrospinal fluid
VDRL: venereal disease research laboratory test
ECDC: European Centre for Disease Prevention and Control
TPPA: Treponema pallidum particle agglutination assay
FTA: abs-fluorescent treponemal antibody absorption
CDC: Centers for Disease Control and Prevention
